# Design of a printed electrochemical strip towards miRNA-21 detection in urine samples: optimization of the experimental procedures for real sample application

**DOI:** 10.1007/s00216-023-04659-x

**Published:** 2023-03-31

**Authors:** Wanda Cimmino, Davide Migliorelli, Sima Singh, Antonella Miglione, Silvia Generelli, Stefano Cinti

**Affiliations:** 1 Department of Pharmacy, University of Naples Federico II, 80131 Naples, Italy; 2 CSEM SA Centre Suisse d’Electronique Et de Microtechnique, Bahnhofstrasse 1, 7302 Landquart, Switzerland; 3 BAT Center-Interuniversity Center for Studies On Bioinspired Agro-Environmental Technology, University of Napoli Federico II, 80055 Naples, Italy

**Keywords:** miRNA, Screen-printed, Electroanalysis, Cancer, Urine

## Abstract

MicroRNAs (miRNAs) are clinical biomarkers for various human diseases, including cancer. They have been found in liquid biopsy samples, including various bodily fluids. They often play an important role in the early diagnosis and prognosis of cancer, and the development of simple and effective analytical methods would be of pivotal importance for the entire community. The determination of these targets may be affected by the different physicochemical parameters of the specimen of interest. In this work, an electrochemical detection platform for miRNA based on a screen-printed gold electrode was developed. In the present study, miRNA-21 was selected as a model sequence, due to its role in prostate, breast, colon, pancreatic, and liver cancers. A DNA sequence modified with methylene blue (MB) was covalently bound to the electrochemical strip and used to detect the selected target miRNA-21. After optimization of selected parameters in standard solutions, including the study of the effect of pH, the presence of interferent species, and NaCl salt concentration in the background, the application of square-wave voltammetry (SWV) technique allowed the detection of miRNA-21 down to a limit in the order of 2 nM. The developed device was then applied to several urine samples. In this case too, the device showed high selectivity in the presence of the complex matrix, satisfactory repeatability, and a limit of detection in the order of magnitude of nM, similarly as what observed in standard solutions.

## Introduction

Prostate cancer (PCa) has an incidence of more than 1.2 million new cases and 350,000 deaths yearly [1]. Statistics report that PCa is the second most common cause of cancer mortality in men globally [2]. The chances of occurrence increase significantly with age, with > 85% of newly diagnosed individuals are over 60 years old [3]. The outlook for distant metastatic PCa (mPCa) is poor, and the overall 5-year survival rate is only 31% [1]. Therefore, diagnosis in the initial stage of the disease, prior to the development of metastatic sites, is vital to reduce mortality and to the successful onset of treatment.

Regular monitoring of prostate-specific antigens (PSA) and digital rectum examinations (DREs) are the gold standard screening technique for prostate cancer in clinical practice [4–6]. However, the precision of these approaches for detecting PCa is limited. PSA analysis has low specificity, resulting in a high risk of false positives or negatives. Biopsies are unpleasant processes that can result in infections, incontinence, and erectile dysfunction. Moreover, it has not been possible to this date to specify a precise range of PSA levels in mPCa [7].

Additionally, DREs are subjective tests and the degree of precision depends on the examiner's experience [8]. In a meta-analysis, DRE has shown a sensitivity and a positive predictive value (PPV) of 17.8% for PCa [6]. Recently, there has been an upsurge in liquid biopsies that use circulating tumor cells, exosomes, DNA, RNA, or microRNAs (miRNA) as biomarkers. Liquid biopsies are gaining ground as routine, minimally invasive tests for regular monitoring, early diagnostics, and monitoring of chronic and recurring diseases [9–14]. Research shows that miRNAs play an important role in the development of various diseases, including oncogenesis and metastasis [15–17]. Some type of miRNA control gene expression post-transcriptionally by repressing or degrading mRNA transcripts [18]. The presence of specific miRNA in various bodily fluids makes them circulating biomarkers of high interest for the research in the field of liquid biopsy [19].

Recent observations indicate that a “signature” of determined groups of microRNA widely influence pathways associated with tumor development, progression, and response to therapy [20]. MiRNAs can thus act as fingerprints to detect disease, making them a viable biomarker. For example, ongoing studies focused on lung cancer, blood miRNAs associated with individual risk stratification by low-dose computed tomography (LDCT) are currently object of a large clinical trial (> 4000 participants) at the Multicentric Italian Lung Detection (MILD). The study demonstrates that the combination of a prespecified circulating miRNA signature and LDCT improves the prediction of individual lung cancer incidence and mortality over LDCT alone [21]. In the field of breast cancer, large trials have been designed using multi-gene expression to personalize the therapies, including OncotypeDX, TAILORx, and RxPONDER [22–24]. These trials could allow in future to address specific markers of response, facilitating tumor eradication and limiting excessive therapies. In addition, the scientific community is now considering the relevance of miRNA (both tumor and circulating) to be added to the markers’ list for triple-negative breast cancer, as reported in different studies [24]. The effectiveness of circulating miRNAs as diagnostic biomarkers for the precise and early diagnosis of PCa has been reported in multiple studies [25–27]. Even if not validated by massive clinical trials, many recent studies have reported promising findings relative to using miRNAs for future diagnostic/prognostic applications.

So far, considerable effort has been made to develop sensitive and selective analytical methods for detecting miRNA. The main traditional assays developed to analyze miRNA molecules include real-time polymerase chain reaction (RT-PCR) [28], Northern blot [29], in situ hybridization (ISH) with locked nucleic acid probes [30], next-generation sequencing (NGS) [31], fluorescence in situ hybridization (FISH) [32], and microarrays [33]. These techniques tend to have limitations such as the requirement of qualified manpower, being time-consuming, and the need to access to laboratory facilities with costly high-end instruments [34]: those may include the isolation of target, gel denaturation, transfer to external/solid support (blotting), and the use of specific equipment and tools. A cost in the range of ca. 1000 Eur is associated to these approaches, and this does not include the cost of equipment. In order to overcome limitations caused by laboratory-bound approaches, a wide range of sensor- and biosensor-based techniques can be used, based on colorimetry, fluorescence, based on cantilevers, and electrochemical. For example, Choi and Seo reported a hairpin structure-mediated diagnostic method for the simple and rapid colorimetric detection of miRNA with high sensitivity (LOD = 132 aM) and high selectivity [35]. Nemati and Hosseini detected miRNA by an enzyme-free fluorescence probe with a 5 pM detection limit [36]. Recently, Andrade et al. reported a nanomechanical genosensor based on an atomic force microscope (AFM) cantilever functionalized with complementary strands of miRNA-203 and miRNA-205 which are potential biomarkers of metastasis in head and neck squamous cell carcinomas (HNSCC) [37]. Although these methods showed an acceptable range of accuracy and precision in detecting miRNA in biological samples, electrochemical architectures are promising, due do their miniaturization potential, simple experimental setup, and direct application towards colored matrices, where colorimetric methods often meet their main limitation. In addition, they can be designed for multiplexed measurements [38, 39]. The adoption of miniaturized electrochemical sensors, exploiting the advantage of microfabrication, and the use of hybrid nanoprobes have demonstrated to be satisfactorily applied towards the detection of clinically relevant molecules, i.e., exosomes, protein, nucleic acids, and enzymes [40]. Several different electrochemical architectures have been reported to detect circulating miRNAs: for example, miRNA-492 has been detected in serum samples through the nanoengineering of a paper-based electrode with a peptide nucleic acid (PNA) probe that was coupled to an external ruthenium-based redox probe, producing a current signal [41]. A more sophisticated approach was developed exploiting DNA nanotechnology, with a novel concept of dynamic nanomachine, defined as “walking and rolling.” This approach is powered by two enzymes, namely nuclease and nicking endonuclease, and the presence of pH-controlled triplex structures, which allows the reversible use of the nanomachine. The main drawback of this approach is that several steps are necessary. Authors achieved an ultra-low detection limit in the order of 10^−18^ M [42]. A different strategy, based on the formation of a miRNA/DNA assembly was used to detect miRNA sequences, that was electrochemically quantified by observing the hydrogen peroxide reduction in the presence of HRP-labeled p53 and hydroquinone [43]. Another novel and sophisticated strategy to detect miRNA-141 uses the RNA-triggered copper ion reduction method. Authors reported the possibility to detect a miRNA target in the presence of DNA recognition strand, dithiothreitol, and reducing agents: briefly, the presence of target miRNA induces the reduction of Cu^2+^ with a subsequent change in electrochemical signals generated from the remaining Cu^2+^ [44]. Although different approaches have been developed, it should be noted that some of these are based on complex and time-consuming procedures, often involving the use of external reagents to carry out the assay. In addition to this, the reported application to real samples is scarce. Test in real complex biological media are of high relevance to understand the drawbacks of a technique and evaluate troubleshooting approaches. Relatively to prostate cancer, a recent preliminary study highlighted the possibility of discriminating prostate cancer patients from healthy controls by detecting a signature of miRNAs, including miRNA-21 [45], and urine seems to be a promising matrix to be investigated [46]. The electrochemical platform herein reported (to be also adapted to other circulating miRNAs) represents an easy-to-use and quick cheap device that does not require skilled technicians and/or equipment like for the RT-qPCR. In particular, the adoption of RT-qPCR is also not suitable for portable screening tool, considering RNA is first transcribed into complementary DNA by reverse transcriptase, and the use of primers and proprietary instruments are necessary for the quantification.

According to these findings, we report in the present study the development of a screen-printed electrochemical platform for the determination of miRNA-21 in real urine samples. The portable sensing platform, to be used in decentralized settings, has been developed on gold-based screen-printed electrodes. The herein reported work describes the detailed fabrication procedure of the disposable platform, which uses a methylene blue tagged immobilized DNA probe, providing an integrated sensing platform without the necessity of additional/time consuming tasks to carry out the determination. The study focuses in particular on the effect of pH and salt concentration and their effect on the sensitivity of the platform. The optimized probes and salt concentrations were then applied to a preliminary study in real urine samples. According to the results, miRNA-21 has been detected down to a low nanomolar range with high selectivity. The reported results open the possibility of developing multiplex screen-printed devices for miRNA signature determination in heterogenous urine samples and the use of such samples for liquid biopsy purposes.

## Experimental section

### Materials and apparatus

Sodium chloride (NaCl), sodium hydrogen phosphate (Na_2_HPO_4_), magnesium chloride (MgCl_2_), 6-Mercapto-1-hexanol (MCH, C_6_H_14_OS), and tris(2-carboxyethyl)phosphine (TCEP; C_9_H_15_O_6_P) were purchased from Sigma-Aldrich (St. Louis, MO, USA). The MB-DNA probe selective to miRNA-21 (5′thiol C6 SS-TCAACATCAGTCTGATAAGCTA- methylene blue 3′) and the miRNA-21 target (5′UAGCUUAUCAGACUGAGUUGA 3′) were purchased from LGC Biosearch technologies, Hoddesdon, UK. Thin-film gold electrodes (ref. DRP-PW-AU10) were purchased from Metrohm DropSens. Deionized water (DI) was used to perform the experiments. All solvents and chemicals were of analytical grade. The urine samples have been collected directly from volunteers working at CSEM SA—Center Landquart. Electrochemical measurements were performed with a potentiostat MultiPalmSens 4, 8 channels (PalmSens, the Netherlands), and data were recorded with potentiostat MultiPalmSens software PsTrace interfaced with a laptop.

### Sensor fabrication on screen-printed gold electrodes

**Fig. 1:**
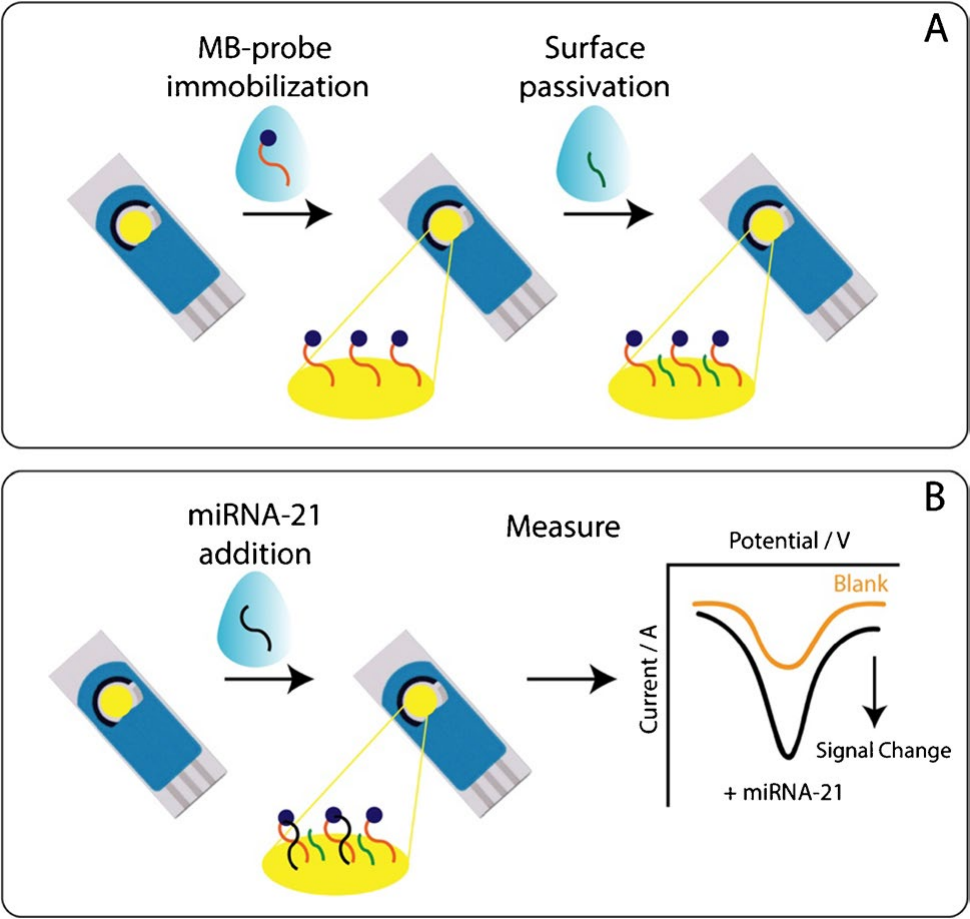
**A** Schematic representation of electrochemical device fabrication and **B** principle of miRNA-21 detection

Before the immobilization of the probe, the working electrode surface was cleaned by following a protocol reported by the research group of Plaxco [47].To do this, we first performed a series of cyclic voltammetry (CV) cycles in 0.5 M H_2_SO_4_, scanning from 0 to + 1.5 V (step of 1 mV at a scan rate of 1 V/s), until the gold peak became stable (approximately 20 scans). After thorough rinsing with DI water, the active area of the electrode was dried, avoiding contact with the three-electrode system. After this, the immobilization of the oligonucleotide probe was carried out in the following three main steps [48]: (1) the 100 μM MB-DNA probe, dissolved in 50 mM phosphate buffer pH = 7, 250 mM NaCl, 10 mM MgCl_2_, was first reduced in the presence of 0.01 M TCEP for 1 h prior to being immobilized; (2) the resulting solution was diluted in 50 mM phosphate buffer to the chosen concentration (in the nanomolar range) for immobilization on the screen-printed working electrode. To do this, 6 μL of the diluted solution was added onto the working electrode for 1 h in a humidity chamber at room temperature. Subsequently, the working electrode surface was carefully rinsed with DI water. Finally, (3) the working electrode was incubated with 6 μl of 2 mM MCH in a humidity chamber, to passivate the remaining active sites on the working electrode and to define the orientation of the immobilized MB-DNA probes. Before being used, the electrode was rinsed with DI water to eliminate the unbound MCH. The main steps for modification are reported in Fig. 1A.

### Measurement of miRNA target

Our electrochemical measurements for the miRNA target were performed at room temperature with eight electrodes inserted into a multi-eight potentiostat connected to a laptop. Square wave voltammetry (SWV) was performed using the following parameters (T equilibration = 5 s, E begin = 0.1 V, E end = − 0.4 V, E step = 0.001 V, amplitude = 0.04 V, frequency = 5–1000 Hz). Briefly, the sensing architecture is based on the affinity between the miRNA-21 and the immobilized probe. When the target is recognized at the modified surface of the electrode, the signal due to the electron transfer, mediated by methylene blue tag, is affected. The signal change is correlated with the presence of miRNA-21 that is hybridized with the complementary DNA probe that is immobilized on top of the working electrode. The signal change is based on changes in electron transfer as a function of reporter separation from the electrode surface. From a physical point of view, two populations of probes exist on the electrode with different rates of electron transfer, one slower than the other. Because the rates are different, the time constants, i.e., half-life, of the current decay after pulsing the voltage will be different for each of the two populations. By adjusting the frequency of the square wave, the frequency of measurements is tuned to the time constant of the electron transfer process for the populations. When the high frequency is tuned to the time constant of the folded population, it increases upon target binding, and the consequence is the possibility to have both ON and OFF responses. With hybridization systems, the oligo-duplex may be so rigid that is not possible to experimentally match the time constant of the electron transfer event (low frequency). To our knowledge, in literature the existence of signal OFF and signal ON frequencies has been described towards the detection of the target analyte [49].

The modified electrode was stabilized for 30 min in a buffer solution before detecting the target. After 30 min, the signal of the immobilized probe was considered stable and the background signal (blank) was registered (Fig. 1B). Following the background detection, the sample solution containing the miRNA target in the nanomolar range was added to the surface of the sensor, and after 20 min, the signal was recorded. For in the determination of the calibration curve, the % signal change I_%_ was plotted against the concentration of the target analyzed. With the use of the following equation, we were able to calculate the percentage of signal change for a given frequency:

1 $${I}_{\mathrm{\%} }=\left(\frac{{I}_{target}-{I}_{0}}{{I}_{0}}\right)*100$$

where *I*_target_ represents the current signal obtained in presence of miRNA-21 and *I*_0_ represents the current signal obtained in the absence of target.

## Results and discussion

### Optimization of experimental parameters in standard solution

All the experimental parameters have been optimized to develop a biosensor adapted to detect miRNA-21 in real urine matrix with minimal sample preparation. The electroanalytical platform is based on a binding-induced conformational change of an immobilized oligonucleotide probe containing a redox reporter, in the present case methylene blue. When the target is present in the solution, it leads to a change in electron transfer that is quantified by using square-wave voltammetry. The optimization was performed in 50 mM phosphate buffer solutions pH = 7 (containing 10 mM MgCl_2_) and different concentrations of sodium chloride, depending on the experimental conditions. The adjustment of ionic strength was achieved by variation of sodium chloride concentration. The signal change in function of the frequency has been calculated from the data collected by varying the square-wave frequency between 5 and 1000 Hz. These electrochemical architectures can be divided into “signal on” and “signal off,” respectively. The presence of the target increases and decreases the peak current [50–52]. In the present case, we observe a”signal on” behavior.

It should be noted that an electrostatic repulsion exists between negatively charged target and probe. In such case, high ionic strength is required for the successful binding of the two molecules [53]. To perform these studies, the final configuration of the working electrode was obtained by modifying it with a 250-nM probe solution that gave the best compromise in terms of sensitivity and signal-to-noise ratio. In fact, 125 and 500 nM were also tested for the manufacturing of the device, but the former was consistent with a low amount of immobilized probes, giving low current, and the latter resulted in a too high concentration of probe on the sensor surface, creating a steric hindrance which affected probe-target recognition and resulting in low signal change.

**Fig. 2:**
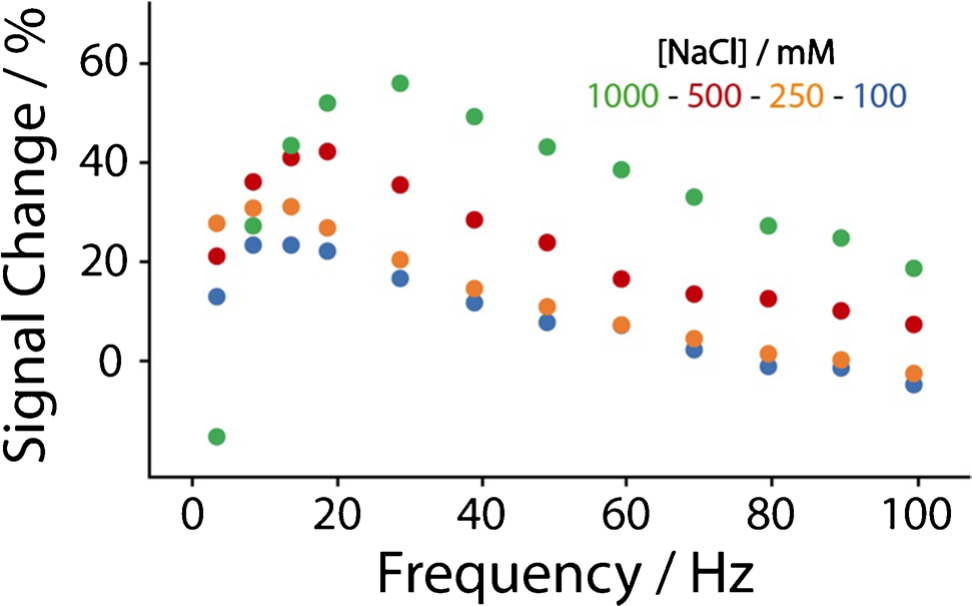
Evaluation of the correlation between square-wave frequency and signal change using different concentrations of sodium chloride in 50 mM phosphate buffer pH = 7 (containing 10 mM MgCl_2_), namely 100 (blue dots), 250 (orange dots), 500 (red dots), and 1000 mM (green dots). All the studies have been performed in triplicate, the reported value is the mean of measurements (standard deviation < 10%, not shown), and the tested concentration of miRNA-21 was of a nominal value of 60 nM

**Fig. 3:**
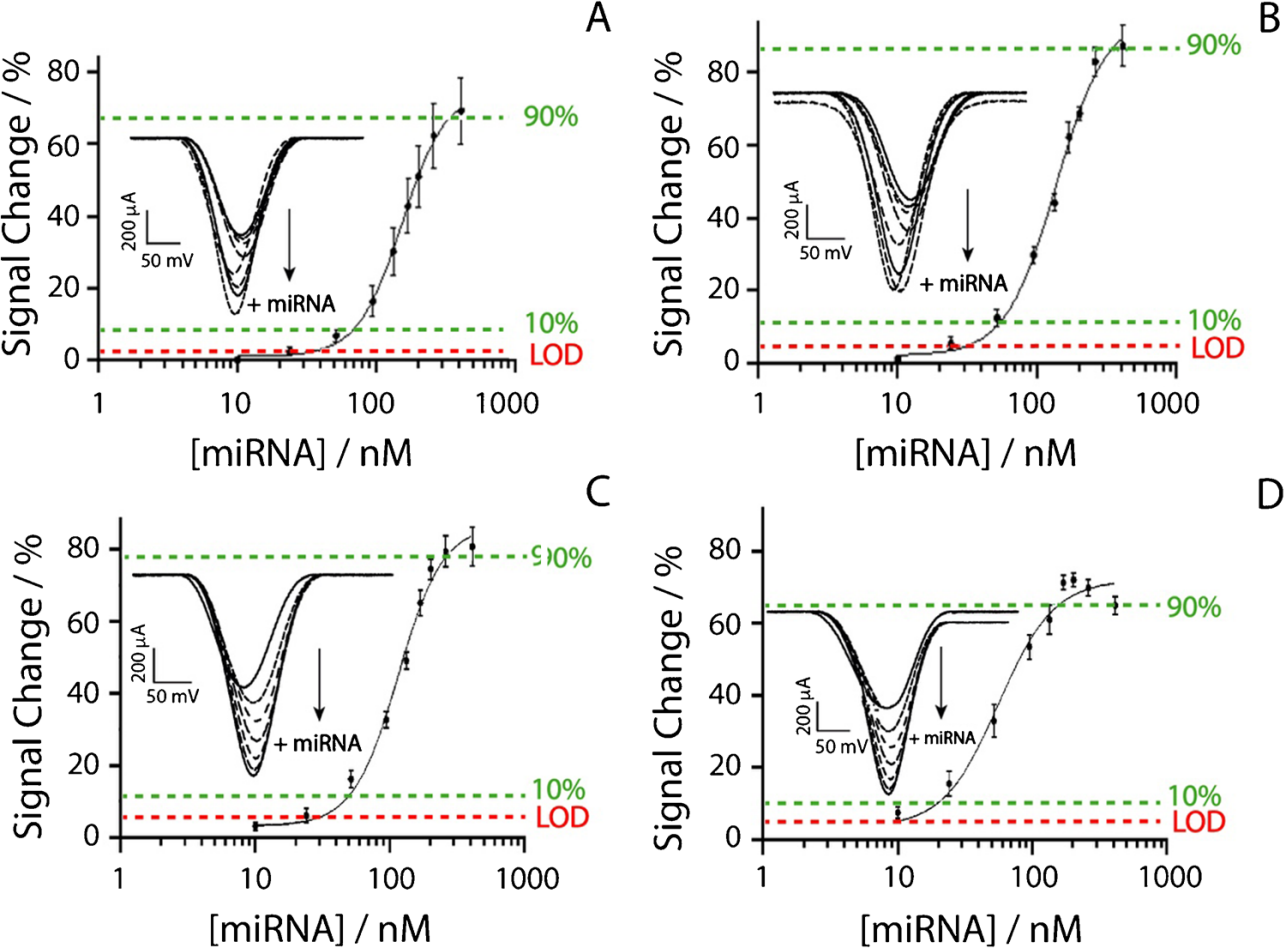
Binding curves obtained at 15 Hz in presence of miRNA-21 ranging from 10 to 410 nM in 50 mM phosphate buffer solution spiked with **A** 100, **B** 250, **C** 500, and **D** 1000 mM of sodium chloride. Insets of each figures display the “signal on” voltametric curves responding to the increase of miRNA-21. All the studies have been performed in triplicate

**Fig. 4:**
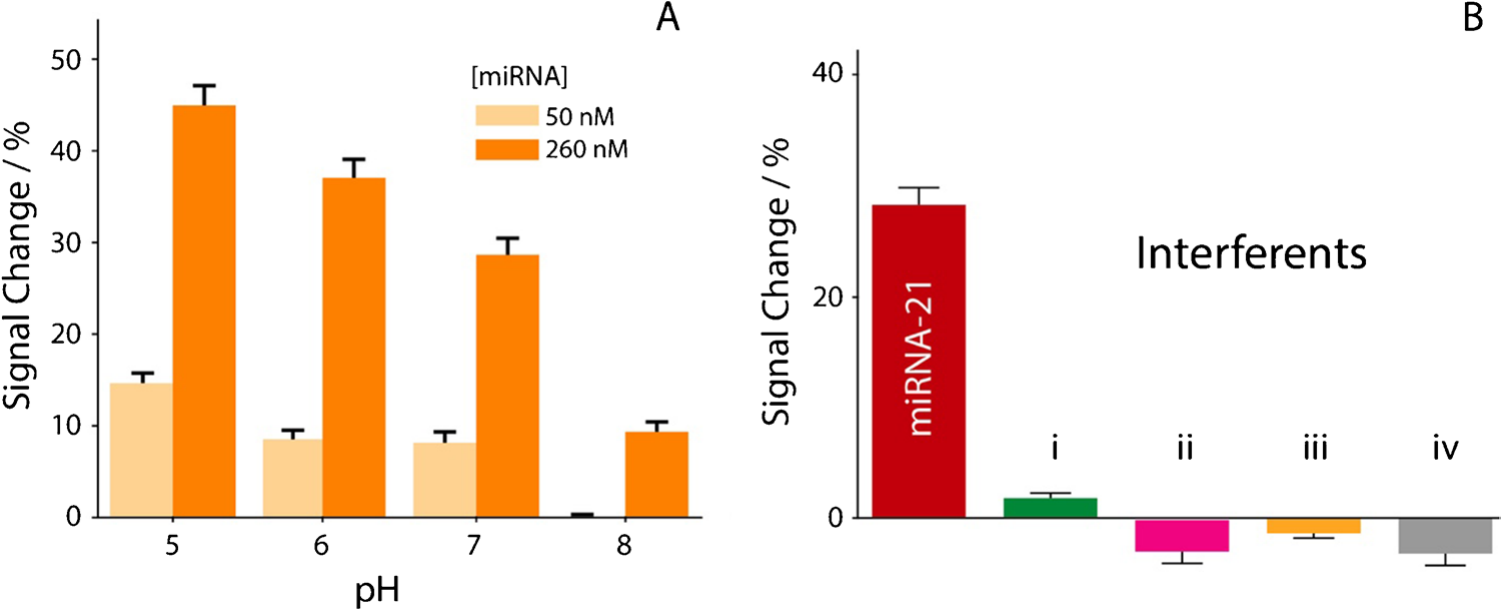
**A** Evaluation of the effect of pH in buffer solutions spiked with 250 mM sodium chloride. The effect of pH was investigated ranging from 5 to 8, using two fixed level of miRNA-21, namely 50 nM (light bars) and 260 nM (dark bars). **B** Selectivity studies in buffer solutions, comparing the signal intensities obtained in the presence of 50 nM miRNA-21 (red bar) and in the presence of two RNA single strands (i, ii) and two DNA single strands (iii, iv). All the studies have been performed in triplicate

**Fig. 5:**
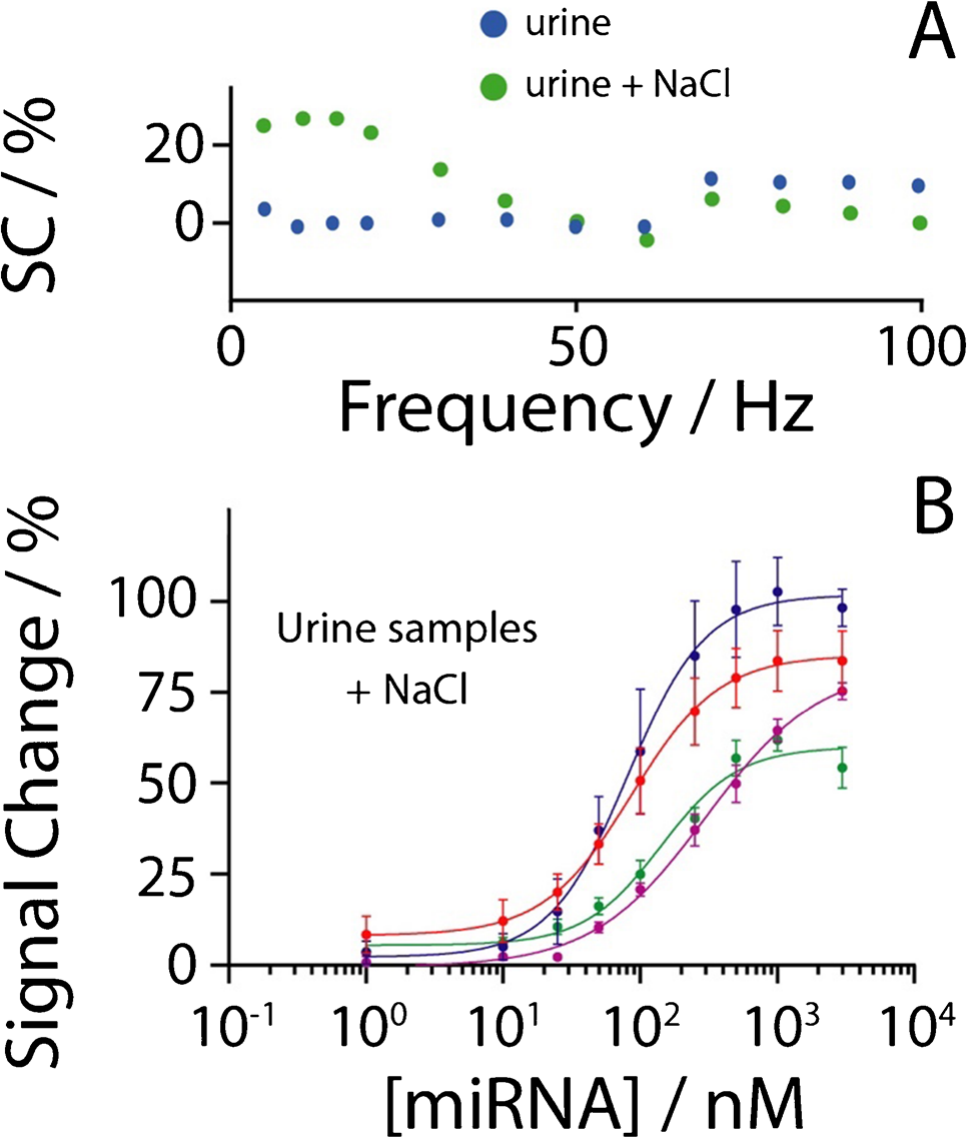
**A** Effect of the addition of 250 mM sodium chloride in urine sample application. **B** Binding curves in the presence of miRNA-21 ranging from 10 to 3000 nM in four different urine samples with the addition of sodium chloride. All the studies have been performed in triplicate

For these experiments, 50 mM phosphate buffer at pH equal to 7 was used as the working solution to which the following concentrations of NaCl were added: 100, 250, 500, and 1000 mM. For all the experiments, a concentration of 60 nM miRNA-21 was used. As shown in Fig. 2, the device is characterized by a “signal on” change, and a signal change increase can be observed as the ionic strength increases. This is ascribable to the higher sodium concentration, which promotes probe-target recognition. In addition, the higher signal change is associated with low square-wave frequency in the 10–30 Hz. Among these, the signal at 15 Hz represented a good compromise in terms of sensitivity and repeatability. The frequency tests have been performed up to 1000 Hz; however from 100 to 1000 Hz, signal change was not significant. By consequence, the frequency of 15 Hz was chosen to analyze various concentrations of miRNA-21 in buffer solutions and in urine, as reported in Figs. 3, 4, and 5.

All the experiments have been carried out in the presence of miRNA-21 ranging from 10 to 410 nM, utilizing 15 Hz as the square-wave frequency and varying the concentration of sodium chloride from 100 to 1000 mM. Depending on the different concentrations of sodium chloride, each platform was able to detect miRNA-21 down to 34, 31, 30, and 8 nM, respectively, adding 100, 250, 500, and 1000 mM to the buffer solution. The dynamic ranges were calculated for each set of parameters as the target concentration which produced a signal change comprised between 10 and 90% of the maximum: the use of 100, 250, 500, and 1000 mM sodium chloride produced the dynamic ranges of 70–345 nM, 50–340 nM, 50–270 nM, 20–160 nM, respectively. Interestingly, when 250 and 500 mM sodium chloride were added, the curves showed very similar performance in terms of detection limit and repeatability (ca. 10%, calculated as the relative standard deviation). All the analytical features have been included in following Table 1.

**Table 1:** Analytical performance of the developed e-platform for miRNA-21 detection

**NaCl level (mM)**	**LOD (nM)**	**Dynamic range (nM)**	**CV (%)**
100	34	70–345	15
250	31	50–340	8
500	30	50–270	9
1000	8	20–160	12
**Recovery studies**			
**Added (nM)**	**Founded (nM)**		**Recovery (%)**
50	45		90
100	105		105

Considering that the physiological range of sodium chloride in the urine ranges in general from 20 to 250 mM, and based on the abovementioned results, it was decided to proceed with the analysis in urine by supplementing the raw samples with an additional amount of sodium chloride to reach exogenous 250 mM, in order to reach a NaCl level comprised between 250 and 500 mM.

### Evaluation of the pH effect and interferents

The effect of the variation of pH was investigated, because for real matrix application in urine, the pH might be diverse among the various samples, varying in general from pH 4.6 to pH 8 [54]. In particular, a high pH value (generally > 8.5–9) can lead to the denaturation of the probe/target double helix. In contrast, a low value of pH (< 3) can lead to the hydrolysis of the nucleic acid strands [55]. The effect of the pH on the sensing architecture was evaluated by varying the pH of the sensing buffer solution between 5 and 8, as reported in Fig. 4A.

All the experiments were performed using the optimized experimental conditions, and for each pH value, the concentrations of miRNA-21 interrogated were 50 and 260 nM. pH 5 gave the best results in signal variation. The next step of investigations was to evaluate the response of the platform in the presence of interference. For this study, the device was interrogated in the presence of four interferents, as reported in inset of Fig. 4B. The selectivity of the platform was tested in the presence of the following single-stranded RNA and DNA targets: (i) 5′-AAACUUUUGGGGAUGACGA-3′, (ii) 5′-ACACUUCCGCGCGAUGACGCC-3′, (iii) 5′-TATCCCATTTAGACTACTA-3′ and 5′-TATCCATTAGACTACTACGCA-3′. All the experiments were performed in the presence of 50 nM of the sequences described above, and they were compared with the detection of 50 nM of the entirely complementary miRNA-21 target (5′-UAGCUUAUCAGACUGAGUUGA-3′). The presence of interferents was only responsible for neglectable signal changes, lower than 10%.

### Urine samples monitoring of miRNA-21

The following steps of the work were focused on the analysis of miRNA-21 in urine samples. From the results of the studies in standard solution, it was defined to adjust the salt content of real urine samples by an additional 250 mM NaCl. As shown in Fig. 5A, the square-wave frequency studies (up to 100 Hz) highlighted how, for these samples, the addition of 250 mM of sodium chloride was effective in inducing a detectable and reproducible signal change, producing a similar trend as obtained in standard solutions.

Subsequently, four different urine samples were spiked with miRNA-21 ranging from 1 to 3000 nM for a preliminary evaluation of the applicability of the platform when in use with this complex medium. For each urine sample, as resulting from the optimization tests, a 250-mM NaCl solution was added, in order to achieve a background total concentration of sodium chloride ranging between 250 and 500 mM. The pH was measured prior to perform miRNA-21 quantification. As displayed in Fig. 5B, four sigmoidal curves were obtained for the four urine samples tested. It should be noted that the pH value was slightly different, namely 6.38, 6.22, 6.50, and 7.20, respectively. The adoption of exogenous sodium chloride allowed satisfactory results in the presence of miRNA-21 in the range between 10 and 3000 nM, as displayed in Fig. 5D, reaching detection limits down to 10, 34, 2, and 24 nM. It should be noted that all the real urine samples have not been treated, causing the different LOD values that have been calculated; however, the analysis of different pH- and ionic strength samples might be mitigated by the use of the standard addition method, thus reducing the effect due to the heterogeneity of untreated samples. These values are in the same order of magnitude with respect to the results obtained in buffer solutions. In addition, with respect to other electrochemical systems reported for miRNA-21 detection in biological samples, Table 2 has been included for a quick comparison, focusing on the platform, the analytical features, and the experimental setup.

**Table 2:** Comparison of the proposed miRNA-21 platform with other electrochemical-based approaches

Platform	LOD	Matrix	Required tasks	Ref
LNA-integrated nucleic acid hairpin probe, biotin-labeled bridge DNA–AuNPs–bio-barcode, enzymatic signal amplification	6 fM (buffer)	Human hepatocarcinoma cells extract	Addition of HRP, H_2_O_2_, hydroquinone	[56]
ssDNA/pSAM/ITO recognized RNA, and ALP-conjugated JAZ binds RNA–DNA hybrid	30 fM	tenfold diluted serum	Cell stored for 10’ at 37 °C in TZ buffer with Ru(NH_3_)_6_^3+^, HQDP, TCEP	[57]
Sulfonamide-bound antisense hybridization	20 fM (buffer)	Urine after proteinase K digestion by spin-filtration	30’ shaking at 50 °C, addition of external electrochemical probe (ferricyanide)	[58]
Magnetosensing platform using p19 viral protein as receptor	0.45 nM (buffer)	Breast cancer cells Extract	Magnetic beads, HRP, H_2_O_2_, 75’	[59]
miRNA-DNA-MB hybridization onto AuNPs	2 nM	Urine	Addition of NaCl	This work

*LNA*, locked nucleic acid; *ssDNA*, single stranded DNA; *pSAM*, polymeric self-assembled monolayer; *ITO*, indium − tin oxide; *ALP*: alkaline phosphatase; *JAZ*, just another zinc finger protein ZNF346; *HRP*, horseradish peroxidase

With respect to other existing methodologies, it should be noted that the approach which involves the use of a MB-modified recognizing probe represents a very easy-to-use approach, based on the fact that all the reagents are already incorporated on top of the working electrode. Although other systems are characterized by lower detection limit, down to fM level (often based on measurements carried in buffer solutions), it should be highlighted how these approaches are based on both laborious manufacturing and measuring tasks, for instance, involving the addition of external electrochemical mediators, enzyme–substrate, magnetic beads for pre-concentration, and in some cases heating steps are performed. These systems are majorly developed using traditional/bulk electrodes, and the adoption of such disposable screen-printed platform makes the proposed device very interesting for following application, also considering the unique application in un-treated urine.

## Conclusion

Timely diagnosis and effective therapies are essential to improve the chances of survival for PCa patients. To enable point-of-care testing, analytical devices need to satisfy the ASSURED principle established by the World Health Organization. Herein, we reported the development of an electrochemical printed platform to determine miRNA-21 making use of a DNA probe. After optimization of the experimental parameters in standard solutions, the feasibility of analysis of miRNA-21 in real urine samples has been evaluated. The present study was launched with the target of finding novel solutions for the design of portable health screening and diagnostics methods for the application to real urine samples. We demonstrated the potential of the method for simple detection assays in complex bodily fluids. The developed devices detected miRNA-21 down to 2 nM in real urine samples. When real matrices are interrogated, the major drawback is represented by the heterogeneity among patients; with this study we evaluated various strategies to compensate for it. The impact of multiple treatment procedures on the detection of the target, i.e., salt concentration and pH adjustment, was discussed. The results of this study indicate that the present approach represents a valid starting point toward the future development of multi-sensory platforms, possibly in combination with multivariate analysis/artificial intelligence, to detect validated miRNA signatures for ambulatory health screening tests and monitoring for early detection of tumor or its recurrence. In addition, these results could be implemented in future by combining the fully integrated printed platform with additional steps, i.e., magnetic beads, aimed to improve the sensitivity of the method, even if these steps would be consistent with an increase of the complexity of the assay.
